# Response Selectivity of the Lateral Posterior Nucleus Axons Projecting to the Mouse Primary Visual Cortex

**DOI:** 10.3389/fncir.2022.825735

**Published:** 2022-02-28

**Authors:** Satoru Kondo, Yuko Kiyohara, Kenichi Ohki

**Affiliations:** ^1^Department of Physiology, School of Medicine, The University of Tokyo, Tokyo, Japan; ^2^World Premier International Research Center – International Research Center for Neurointelligence (WPI-IRCN), The University of Tokyo Institutes for Advanced Study (UTIAS), Tokyo, Japan; ^3^Institute for AI and Beyond, The University of Tokyo, Tokyo, Japan

**Keywords:** lateral posterior nucleus, lateral geniculate nucleus, primary visual cortex, axons, response selectivity, two-photon calcium imaging, mice

## Abstract

Neurons in the mouse primary visual cortex (V1) exhibit characteristic response selectivity to visual stimuli, such as orientation, direction and spatial frequency selectivity. Since V1 receives thalamic visual inputs from the lateral geniculate nucleus (LGN) and lateral posterior nucleus (LPN), the response selectivity of the V1 neurons could be influenced mostly by these inputs. However, it remains unclear how these two thalamic inputs contribute to the response selectivity of the V1 neurons. In this study, we examined the orientation, direction and spatial frequency selectivity of the LPN axons projecting to V1 and compared their response selectivity with our previous results of the LGN axons in mice. For this purpose, the genetically encoded calcium indicator, GCaMP6s, was locally expressed in the LPN using the adeno-associated virus (AAV) infection method. Visual stimulations were presented, and axonal imaging was conducted in V1 by two-photon calcium imaging *in vivo*. We found that LPN axons primarily terminate in layers 1 and 5 and, to a lesser extent, in layers 2/3 and 4 of V1, while LGN axons mainly terminate in layer 4 and, to a lesser extent, in layers 1 and 2/3 of V1. LPN axons send highly orientation- and direction-selective inputs to all the examined layers in V1, whereas LGN axons send highly orientation- and direction-selective inputs to layers 1 and 2/3 but low orientation and direction selective inputs to layer 4 in V1. The distribution of preferred orientation and direction was strongly biased toward specific orientations and directions in LPN axons, while weakly biased to cardinal orientations and directions in LGN axons. In spatial frequency tuning, both the LPN and LGN axons send selective inputs to V1. The distribution of preferred spatial frequency was more diverse in the LPN axons than in the LGN axons. In conclusion, LPN inputs to V1 are functionally different from LGN inputs and may have different roles in the orientation, direction and spatial frequency tuning of the V1 neurons.

## Introduction

In mammals, visual information from the retina flows through two major bottom-up pathways and undergoes hierarchical processing (Nassi and Callaway, 2009). One pathway reaches the primary visual cortex (V1) and subsequent higher visual areas (HVAs) via the lateral geniculate nucleus (LGN) of the thalamus. Another pathway reaches the V1 and HVAs in parallel via the lateral posterior nucleus (LPN) of the thalamus. Both the V1 and HVAs project back to the LPN and form reciprocal circuits with the LPN. The LPN receives strong projections from the superior colliculus (SC) (Gale and Murphy, 2014; Tohmi et al., 2014; Zhou et al., 2017; Bennett et al., 2019), that receives direct retinal input. The V1 sends cortico-collicular feedback projection to the SC and modulate the SC activity (Zhao et al., 2014; Liang et al., 2015), and the SC activity may in turn impact on visual cortical processing via the LPN (Ahmadlou et al., 2018).

LPN is considered the rodent analog of the primate pulvinar (Baldwin et al., 2017; Zhou et al., 2017). LPN/pulvinar receives input from many cortical and subcortical areas, including sensory, motor, association and visuomotor area (Grieve et al., 2000; Wurtz et al., 2011; Gale and Murphy, 2014). LPN/pulvinar combines information from multiple sources and influence the visual processing by linking with the visual, other sensory and the behavioral context (Grieve et al., 2000; Wurtz et al., 2011; Gale and Murphy, 2014; Roth et al., 2016). Previous studies implicated the functional roles of the pulvinar related with higher visual functions in primate, such as attention, saccade movement, and visually guided locomotion (Zhou et al., 2016; Dominguez-Vargas et al., 2017; Soares et al., 2017). In rodent, the LPN transmits motor-related signals to the HVAs and contribute the visual information processing in these cortical area (Tohmi et al., 2014; Beltramo and Scanziani, 2019; Blot et al., 2021), conveys the surround information of the visual field to V1 (Durand et al., 2016; Roth et al., 2016; Fang et al., 2020) and sharpen the orientation selectivity of layer 2/3 neurons in V1 (Fang et al., 2020).

Recent anatomical studies have shown that the LPN is divided into several subregions (Zhou et al., 2017; Bennett et al., 2019) that have specific connections with the cortical and subcortical areas (Tohmi et al., 2014; Bennett et al., 2019). It has been reported that V1 mainly receives projections from the anterior part of the LPN (Bennett et al., 2019), and studies using transsynaptic tracers revealed the bottom-up visual pathway from the SC to V1 through LPN (Fang et al., 2020). In V1, unlike LGN afferents that mainly terminate in layer 4, LPN afferents primarily terminate in layers 1 and 5 (Roth et al., 2016; Zhou et al., 2017). This anatomical segregation of axonal termination in different layers suggests the functional difference between the two thalamic afferents in V1. Among six layers in V1, layer 4 is regarded as the primary input layer of the visual information from the retina (Nassi and Callaway, 2009). On the other hand, layer 1 receives broad range of inputs, such as sensory and higher-order corticocortical, neuromodulatory and thalamic inputs (Mesik et al., 2019). Since the role of cortico-cortical and neuromodulatory inputs to V1 layer 1 has been suggested to be modulatory (Alitto and Dan, 2013; Ibrahim et al., 2016; Leinweber et al., 2017), the role of thalamic inputs to layer 1 may also contribute to the modulation of V1 neuronal activity. However, due to the lack of recordings of the functional properties of the LPN axons distributed in the different V1 layers, the difference in the role of LPN and LGN on the information processing in V1 circuit remains not yet been fully elucidated.

In this study, we performed *in vivo* functional imaging of the LPN axons terminating in various layers in V1 and compared their response selectivity with our previous results on the LGN axons (Kondo and Ohki, 2016). Our studies suggest the differential functional roles of the LPN inputs from LGN inputs on the response selectivity of V1 neurons.

## Materials and Methods

### Animals

C57BL/6 mice (purchased from SLC Hamamatsu, Japan) were used for all experiments. The mice were maintained in an animal facility at the University of Tokyo. The facility housed 2–3 mice per cage in a temperature-controlled animal room with a 12-h/12-h light/dark cycle. All procedures were conducted in accordance with protocols approved by the University of Tokyo Animal Care and Use Committee (approval number: P21-002).

### Local Expression of GCaMP6s in the Lateral Posterior Nucleus

The genetically encoded calcium indicator, GCaMP6s, was locally expressed in the LPN using the adeno-associated virus (AAV) infection method. In some experiments, the LGN was infected with green fluorescent protein (GFP)-expressing AAV, and the LPN, with tdTomato-expressing AAV. AAV2/1-CAG-GCaMP6s, GFP, or tdTomato (∼1 × 10^13^ genome copy/ml; purchased from Vector Core, University of Pennsylvania) was used for the desired expression. Mice were anesthetized with isoflurane (5.0% for anesthesia induction and 1.5% for anesthesia maintenance) and fixed on stereotaxic frames. A skin incision was made at the midline, and the periosteum was removed from the skull. For the AAV injection to the LPN and LGN, a small craniotomy was performed just above the dorsal part of the LPN or the LGN using stereotaxic coordinates (LPN: -1.8 mm from the bregma, 2.0 mm lateral from the midline; LGN: -2.3 mm from the bregma, 2.4 mm lateral from the midline). For the AAV injection to the SC, two small craniotomies were performed above the SC using the stereotaxic coordinates (0 mm anterior from the lambda, 1.0 mm lateral from the midline and 0.5 mm anterior from the lambda, 0.5 mm lateral from the midline). A glass pipette filled with AAV vector solution was inserted (2.4 mm depth from the pia for both the LPN and the LGN, 1.0 mm depth from the pia for the SC) and the AAV solution was injected using either pressure (30–50 nL; Nanoject III, Drummond) or iontophoretic methods (3 μA, 7 s-ON and 7 s-OFF, 3 min; Midgard Precision Current Source, Stoelting). The infection area was typically defined as a circular volume with a 400–700 μm diameter from the injection site. The boundaries of the LPN and the LGN were determined according to the mouse brain atlas (Paxinos and Franklin, 2019).

### Imaging and Visual Stimulation

For imaging the LPN axons, at 2 weeks after infection, a craniotomy (3 mm in diameter) was performed over V1. Next, the dura mater was removed, and a cranial window was constructed by sealing the area with a cover slip. Two-photon calcium imaging of the axons was performed under anesthesia (0.2% isoflurane with 2.5 mg/kg chlorprothixene). Images were obtained using a two-photon microscopy system (A1RMP, Nikon) with a 25 × objective lens (CFI Apo LWD 25XW, NA = 1.10, Nikon) at 920 nm wavelength (MaiTai eHP DeepSee, Spectra Physics). The images were obtained in a 2-D plane at 2 Hz. The image size was 512 × 512 pixels (resolution = 0.125 μm/pixel). The average laser power at the sample was modulated between 10 and 40 mW, depending on the imaging depth. To correct the refractive index mismatch in the brain tissue, the axial position of the objective lens was carefully adjusted to obtain the maximum signal intensity. This improved the resolution of axon imaging, especially of those located deep inside the brain. Layers were initially assigned by Nissl staining in the brain section, layer 1: 0–100 μm, layer 2/3: 100–350 μm, layer 4: 350–450 μm, and layer 5: 450–650 μm. Images were then obtained *in vivo* brain at 50–70 μm below the pia for layer 1, at 200–250 μm for layer 2/3, at 350–450 μm for layer 4, and at 450–550 μm for layer 5.

Visual stimulations were presented on a 32-inch LCD display (ME32B, Samsung) using PsychoPy2 (Pierce, 2008). Orientation selectivity was investigated using drifting square-wave gratings moving in 12 directions [each 30° apart, spatial frequency (SPF) = 0.04 cycles per degree (cpd), and temporal frequency = 2 Hz]. These 12 patterns were presented for ∼4 s each (eight frames), with interspersed gray blank (uniform) stimuli of the same duration. The stimuli were repeated 10 times. For SPF preference analysis, drifting sinusoidal-wave gratings moving in eight directions (0.5 s each) were presented for ∼4 s, with interspersed gray blank (uniform) stimuli of the same duration. SPF took one of six different values (0.01, 0.02, 0.04, 0.08, 0.16, and 0.32 cpd) and the temporal frequency was 2 Hz.

### Histology

After imaging, each mouse was transcardially perfused with 4% paraformaldehyde, and the brain was removed and submerged in the fixative solution overnight. Thereafter, coronal sections were obtained (thickness of either 50 or 100 μm), which were subsequently examined localization of the infection site in the LPN or LGN and the distribution of LPN or LGN axons in V1. For assigning the V1 layers, the sections were stained using NeuroTrace Red (cat# N-21482, Molecular Probes). All fluorescent photographs were obtained using an epifluorescence microscope (BZX-710, Keyence). The photographs of the retrogradely labeled areas were taken by either the optical sectioning method with structured illumination (BZX-710, Keyence) or confocal microscope (A1R HD25, Nikon).

### Data Analysis

All analyses were performed using custom-written programs in MATLAB (Mathworks). Data from the different experimental groups were processed using the same computer code; thus, randomization and blinding were not necessary for data analysis. To obtain an orientation map, calcium signal changes were calculated for all the pixels, and each pixel was colored according to the response to the orientation stimulation [hue: preferred orientation; lightness: response magnitude; and saturation: global orientation selectivity index (gOSI)].

The LPN boutons were automatically detected by a template-matching algorithm using convolution mask images. The centroids of the boutons were determined from a mask image of the boutons. Time courses of fluorescent change were extracted by averaging a circle around the centroid (radius, 0.5 μm).

To estimate the out-of-focus signal around the individual LPN boutons, ring-shaped masks within 10 pixels (1.25 μm) were made from the edge of the boutons, while excluding the pixels at less than 4 pixels (0.5 μm) from the edge of the boutons to reduce the possible overlap with the signal change of the boutons. If a mask overlapped the neighboring boutons/axons or axonal shafts, then the overlapping area was removed from the mask. The out-of-focus signal obtained from these masks was subtracted from the fluorescence signal of the bouton (contamination ratio = 1.0) (Kerlin et al., 2010).

Orientation selectivity was calculated from the corrected time courses. Visually evoked fluorescent changes were calculated as the change in fluorescence normalized to the baseline fluorescence (dF/F). Baseline fluorescence was obtained from the average of the last four frames during the blank periods. The *p*-value for responsiveness was obtained from the analysis of variance across the blank and stimulus periods. The *p*-value for selectivity was obtained from the analysis of variance across the stimulus periods.

The preferred orientation was calculated using vector averaging (Swindale, 1998), defined by the following equations:


a=ΣR×icos(2θ)i,

b=ΣR×isin(2θ)i,

θ=pref0.5arctan(b/a),

where R_i_ is the response to the ith direction θ_i_ (12 directions; each 30° apart, from 0 to 330°) and θ_pref_ is the preferred orientation.

The gOSI, which is equivalent to 1—circular variance, was calculated using the following formula:


gOSI=sqrt((ΣRsini(2θ)i)2∧+(ΣRcosi(2θ)i)2∧)/ΣR,i

where R_i_ is the response to the ith direction θ_i_ (Wörgötter and Eysel, 1987).

The OSI was calculated using the following formula:


(R-prefR)ortho/(R+prefR)ortho,

where R_pref_ is the response to the preferred orientation and R_ortho_ is the response to the orthogonal orientation to the preferred orientation.

Axonal boutons or neurons were considered responsive when they met the following criteria: *p*-value for responsiveness < 0.001 and maximum response > 3%. Among the responsive boutons or neurons, sharply orientation-selective boutons or neurons were defined when they met the following criteria: *p*-value for selectiveness < 0.001 and gOSI > 0.33.

The preferred direction was calculated using the Gaussian fitting method. A tuning curve was fitted with the sum of two circular Gaussian functions (von Mises distribution), and the peak of the fitting curve was considered as the preferred direction.

The direction selectivity index (DSI) was calculated using the following formula:


DSI=(R-prefR)opp/(R+prefR)opp,

where R_*pref*_ is the response to the preferred direction and R_*opp*_ is the response in the direction opposite to the preferred direction.

Responsive boutons or neurons were defined using the same criteria as those for the orientation analysis. Among the responsive boutons, sharply direction-selective boutons were defined using the following criteria: *p*-value for selectiveness < 0.001 and DSI > 0.3.

The responses to the different SPFs were fitted using the difference of the Gaussian (DOG) function (Hawken and Parker, 1987). The preferred SPFs of the boutons were determined from the peak of the DOG fitting curve. The DOG fittings were evaluated using the R-squared values.

The SPF selectivity index was calculated using the following formula:


(R-maxR)min/R,max

where R_*max*_ is the maximum response and R_*min*_ is the minimum response.

Axonal boutons were considered responsive when they met the following criteria: *p*-value for responsiveness < 0.001 and maximum response > 3%. Among the responsive boutons, the SPF-selective boutons were defined using the following criteria: *p*-value for selectiveness < 0.001, SPF selectivity index > 0.5, and R-squared value > 0.7. Only the selectively responsive boutons were used for further analysis.

### Statistical Analysis

All data are presented as mean ± standard error unless stated otherwise. A two-sided Mann–Whitney *U*-test or unpaired *t*-test was used to compare two independent groups. Analysis of variance (ANOVA) or Bonferroni correction was performed when more than two groups were compared. Statistical significance was set at *p*-value ≤ 0.05 for all the study analyses, except for the definitions of the visually responsive and selective boutons (see “Data Analysis” section). The sample size (n) was defined as the number of mice or images. No statistical analyses were performed to predetermine the sample sizes; the sample size used was similar to that generally employed in the field.

## Results

### Parallel Projection Pathways From the Thalamus to V1

**FIGURE 1:**
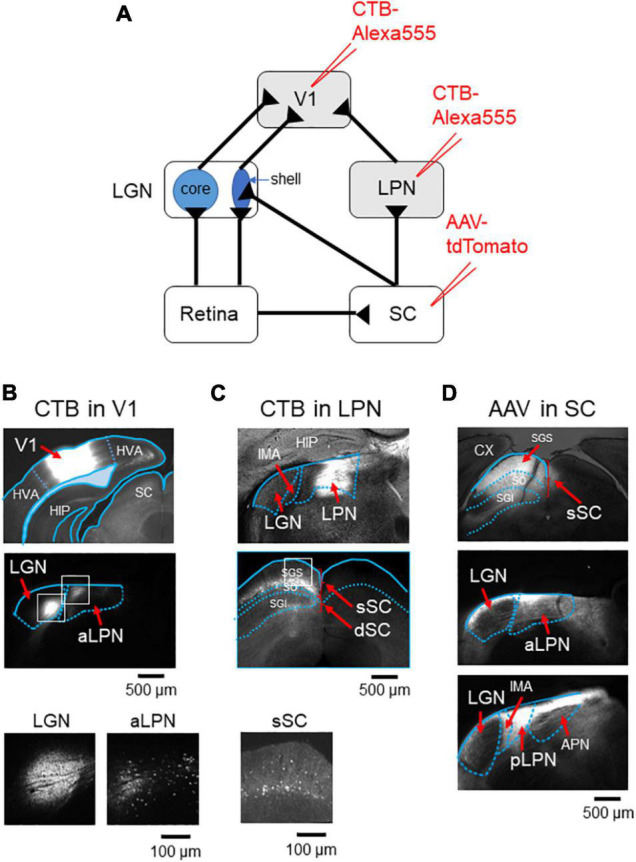
V1 receives inputs from both the LGN and LPN. **(A)** A retrograde labeling by CTB injection into either V1 or LPN. **(B)** CTB was injected into V1 (upper), both the LGN and LPN were retrogradely labeled (middle), and the retrogradely labeled neurons were observed (lower, enlargement of the white rectangles in the middle). **(C)** CTB was injected into the anterior part of the LPN (upper), SC was retrogradely labeled (middle), and the retrogradely labeled neurons were observed (lower, enlargement of the white rectangle in the middle). **(D)** AAV-tdTomato was injected into SC (upper) and both the aLPN (middle) and pLPN (lower) were innervated by SC neurons. Note that the shell of the LGN also received SC inputs (middle). LGN, lateral geniculate nucleus; LPN, lateral posterior nucleus; aLPN, anterior part of LPN; pLPN, posterior part of LPN; CX, cortex; HIP, hippocampus; CTB, cholera toxin subunit B; V1, primary visual cortex; SGS, upper stratum griseum superficial; SO, stratum opticum; SGI, stratum griseum intermedium; sSC, superficial layers of the superior colliculus; dSC, deep layers of the superior colliculus; APN, anterior pretectal nucleus; IMA, intramedullary thalamic area.

Of the two vision-related thalamic nuclei—the LGN and the LPN, it has been suggested that only the LGN receives direct input from the retinal ganglion cells that carry visual information (Allen et al., 2016). To identify the anatomical connections between V1 and LGN or LPN, the retrograde tracer, cholera toxin subunit B (CTB), was injected into V1 (Figure 1A and Supplementary Figure 1A). The CTB injection resulted in retrograde labeling of both the LGN and the anterior part of the LPN neurons, confirming that V1 receives dual inputs from both these thalamic nuclei (Figure 1B and Supplementary Figure 1B). Retinal visual information is divided into two ascending routes that are formed via the LGN or SC. To examine whether the LPN-V1 pathway is different from the LGN-V1 pathway, but related to the SC, CTB was injected into the anterior part of the LPN (Figure 1A and Supplementary Figure 1A), the region from where V1 mainly receives inputs (Figure 1C; Bennett et al., 2019). The CTB injection led to the retrograde labeling of the superficial layer of the SC (sSC) neurons without labeling the LGN neurons, indicating that the LPN-V1 pathway is different from the LGN-V1 pathway and connected to the SC (Figure 1C and Supplementary Figure 1C). Furthermore, to examine the projection of the SC neurons to the LPN and LGN, we anterogradely labeled the SC neurons using AAV-tdTomato (Figure 1D). We confirmed the projection of the SC neurons to the LPN (Figure 1D middle and lower). We observed that the projection from the SC to LPN was denser in the posterior part of LPN (pLPN) (Figure 1D lower) than the anterior part of the LPN (aLPN) (Figure 1D midle) as described previously (Bennett et al., 2019). Furthermore, we noticed that the shell of the LGN where retinal direction-selective inputs innervate also receives inputs from the SC (Figure 1D middle) as reported previously (Bickford et al., 2021). Therefore, the presence of three parallel projection pathways of visual information to V1 were anatomically observed—the LGN (core)-V1 pathway, the SC-LGN (shell)-V1 pathway and the SC-LPN-V1 pathway (Figure 1A).

#### Distinct Laminar Distribution of the Lateral Geniculate Nucleus and Lateral Posterior Nucleus Axons Projecting to V1

**FIGURE 2:**
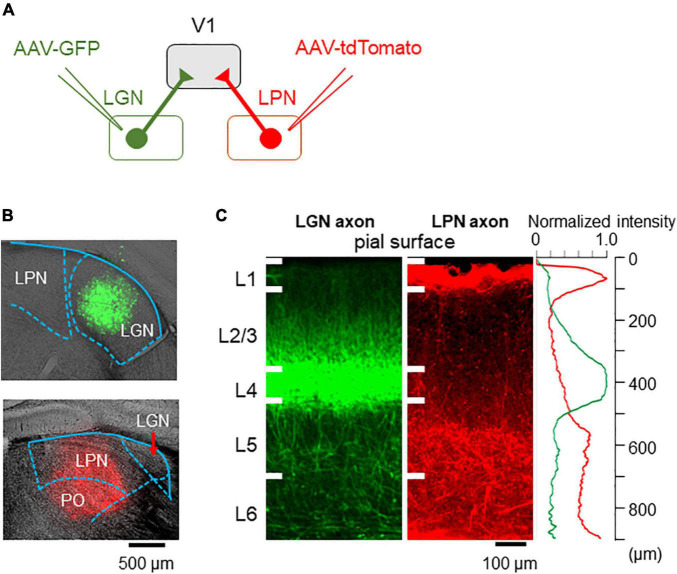
Axonal projections from the LPN and LGN in V1. **(A)** An anterograde labeling of the LGN and LPN axons with different fluorescent proteins. **(B)** A local injection of AAV-GFP in the LGN (upper) and AAV-tdTomato in the LPN (lower). **(C)** The distribution of the LGN (left) and LPN (middle) axons in V1 of the coronal slice. LGN axons project mainly to the layer 4, while LPN axons project mainly to the layer 1 (right). LPN, lateral posterior nucleus; LGN, lateral geniculate nucleus; V1, primary visual cortex; AAV-GFP, adeno-associated virus-green fluorescent protein.

To evaluate whether the two parallel pathways are anatomically segregated or mixed in V1, LGN, and LPN neurons were differentially labeled, and the distribution of the projecting axons to V1 was investigated. AAV-GFP and AAV-tdTomato were locally injected into the LGN and LPN, respectively (Figure 2A). Local injection of the AAV successfully restricted the expression of GFP and tdTomato in the LGN and LPN, respectively (Figure 2B). LGN axons were distributed mainly in the layer 4 and, to a lesser degree, in the layers 1 and 2/3, as previously reported (Figure 2C; Kondo and Ohki, 2016). By contrast, LPN axons were mostly distributed in the layers 1 and 5 (Figure 2C). In the layer 1, LPN, and LGN axons were mixed, however, the relative density of LPN axons was higher than that of LGN axons (Figure 2C). Overall, the parallel LGN and LPN pathways were mostly segregated in the different layers but showed a certain degree of intermingled distribution in V1. With previous work providing evidence that each layer may have a unique functional role in the sensory cortex (Adesnik and Naka, 2018), the results of this study suggest that the two inputs may have different functions in visual information processing in V1.

### Orientation Selectivity of the Lateral Posterior Nucleus Axons in V1

**FIGURE 3:**
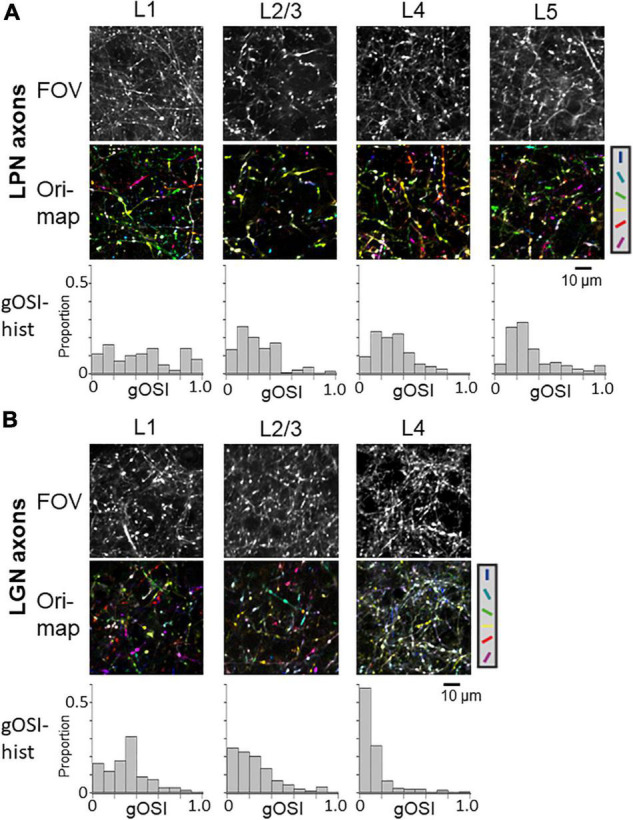
Orientation selectivity of LPN and LGN boutons in V1. **(A)** FOV images (upper), orientation color map (middle) and distribution of gOSI (lower) of the LPN axons in V1. **(B)** FOV images (upper) and orientation color map (middle) and distribution of gOSI (lower) of the LGN axons in V1. Only the LGN axons in the layer 4 indicated low orientation selectivity. LPN, lateral posterior nucleus; LGN, lateral geniculate nucleus; V1, primary visual cortex; FOV, field-of-view. *P ≤ 0.05, ***P ≤ 0.001 The data of LGN axons are re-use from the previous publication (Kondo and Ohki, 2016).

**FIGURE 4:**
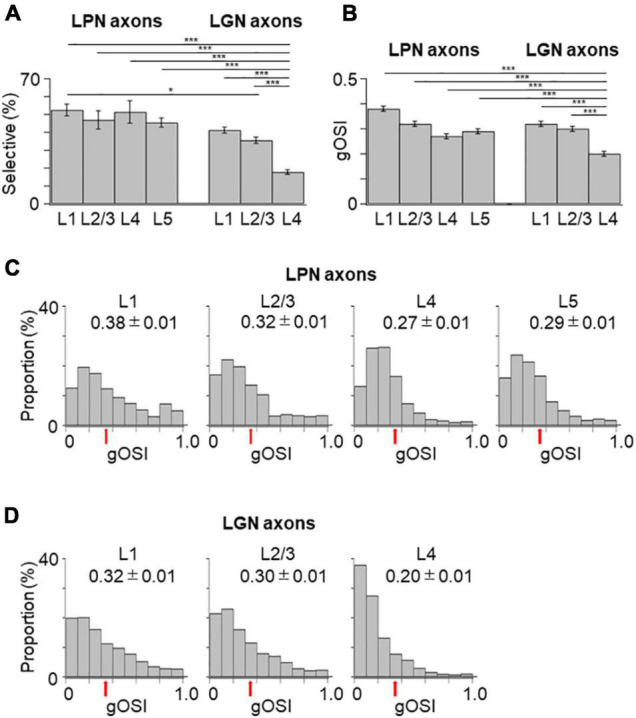
Proportion of orientation-selective axons and distribution of gOSI of LPN and LGN boutons in V1. **(A)** The proportion of orientation-selective LPN and LGN boutons. The proportion of LGN boutons in the layer 4 was significantly different from that of LGN boutons in the remaining layers as well as that of LPN boutons in all the layers (*P* ≤ 0.001, Mann–Whitney *U*-test with Bonferroni correction). The proportion of LGN boutons in the layer 2/3 was significantly different from that of LPN boutons in the layer 1 (*p* = 0.012, Mann–Whitney *U*-test with Bonferroni correction) but no significant difference from layers 2/3, 4, and 5 (*p* = 1, Mann–Whitney *U*-test with Bonferroni correction). The proportion of LGN boutons in the layer 1 was not significantly different from that of LPN boutons in all the layers (layer 1, *p* = 0.118; layers 2/3, 4 and 5, *p* = 1, Mann–Whitney *U*-test with Bonferroni correction). **(B)** The mean gOSI of responsive LPN and LGN boutons. gOSI of LGN boutons in the layer 4 was significantly lower than that of LGN boutons in the layers 1, 2/3, LPN axons in the layers 1, 2/3, 4, and 5 (*P* ≤ 0.001, Mann–Whitney *U*-test with Bonferroni correction). gOSI of LGN boutons between the layers 1 and 2/3 (*p* = 1, Mann–Whitney *U*-test with Bonferroni correction), and gOSI of LPN boutons among different layers were statistically not different (between the layers 1 and 2/3, *p* = 1; between the layers 1 and 4, *p* = 0.105; between the layers 1 and 5, *p* = 0.126; between the layers 2/3 and 4, *p* = 1; between the layers 2/3 and 5, *p* = 0.588; between the layers 4 and 5, *p* = 1, Mann–Whitney *U*-test with Bonferroni correction). **(C)** The distribution of gOSI of LPN boutons in the layer 1 (left, *n* = 562 boutons from six mice, 11 FOVs), layer 2/3 (second from left, *n* = 313 boutons from six mice, 7 FOVs), layer 4 (second from right, *n* = 458 boutons from five mice, 8 FOVs), and layer 5 (right, *n* = 391 boutons from two mice, 9 FOVs). **(D)** The distribution of gOSI of LGN boutons in the layer 1 (left, *n* = 2,924 boutons from 37 mice, 123 FOVs), layer 2/3 (second from left, *n* = 2,818 boutons from 36 mice, 97 FOVs), and layer 4 (second from right, *n* = 1,722 boutons from 30 mice, 98 FOVs). Arrows **(B,C)** indicate the threshold (gOSI = 0.33) for sharply orientation-selective boutons. gOSI, global orientation-selective index; LPN, lateral posterior nucleus; LGN, lateral geniculate nucleus; V1, primary visual cortex; FOV, field-of-view. *P ≤ 0.05; ***P ≤ 0.001. The data of LGN axons are re-use from the previous publication (Kondo and Ohki, 2016).

Previous studies have shown the orientation selectivity of LGN axons distributed in V1 (Kondo and Ohki, 2016; Sun et al., 2016; Zhuang et al., 2021). One study has shown that the projecting axons in the layer 4 have lower orientation selectivity than the projecting axons in the layer 1 (Kondo and Ohki, 2016). However, other studies have reported different results that thalamic axons projecting to both the layers 1 and 4 have high orientation selectivity (Sun et al., 2016; Zhuang et al., 2021). To determine the orientation selectivity of LPN axons projecting to the different layers of V1 and compare their orientation selectivity with that of LGN axons, *in vivo* two-photon calcium imaging of LPN axons was conducted using GCaMP6s. GCaMP6s was locally expressed in the LPN using the AAV method (AAV2/1-CAG-GcaMP6s), and the response to the gratings drifting in 12 different directions was recorded through the cranial imaging window. The fluorescence signal changes in small oval areas, corresponding to the putative axonal boutons, were measured (Glickfeld et al., 2013; Matsui and Ohki, 2013; Kondo and Ohki, 2016). The orientation maps (Figure 3A middle) and the distributions of gOSI (Figure 3A lower) indicated that the selectivity of the LPN axons projecting to V1 was similar among the different layers (Figure 3A, see statistical tests in Figure 4B). In contrast, the LGN axons (Figure 3B middle and lower) indicated lower orientation selectivity in layer 4 than in layer 1 (Figure 3B, see statistical tests in Figure 4B), as previously reported (Kondo and Ohki, 2016).

**FIGURE 5:**
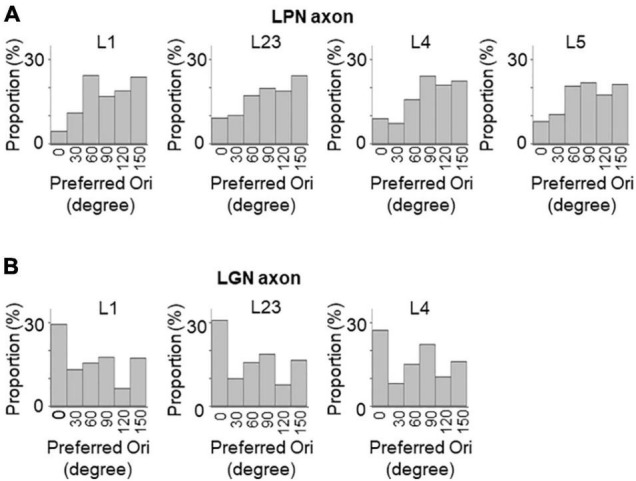
Distribution of preferred orientations of LPN and LGN boutons in V1. The distribution of preferred orientations of LPN boutons **(A)** and LGN boutons **(B)** in each layer of V1. LPN boutons showed skewed bias from horizontal to vertical orientation, whereas LGN boutons showed cardinal bias. LPN, lateral posterior nucleus; LGN, lateral geniculate nucleus; and V1, primary visual cortex. The data of LGN axons are re-use from the previous publication (Kondo and Ohki, 2016).

Next, the orientation selectivity was quantified by calculating the gOSI for both the LPN and LGN bouton populations. The proportion of orientation-selective LPN boutons was not significantly different among the different layers (Figure 4A). However, the LGN boutons in the layer 4 showed significantly lower orientation selective proportion than the other LGN and LPN boutons (Figure 4A). The gOSI of LPN boutons was not significantly different across the different layers (Figures 4B,C). However, the gOSI of LGN boutons in the layer 1 showed significantly higher selectivity than in the layer 4 (Figures 4B,D). Furthermore, analysis of the distribution of the preferred orientations of the LPN and LGN boutons showed that the LPN boutons had a strongly biased distribution of the preferred orientation in all the layers (Figure 5A). The preferred orientations of the LPN boutons showed smaller proportions at 0° (vertical) and 30° than the other orientations. This tendency was similar among LPN boutons recorded from different layers (Figure 5A). The preferred orientation of the LGN boutons showed a weak bias toward cardinal orientations (Figure 5B), as previously reported (Kondo and Ohki, 2016). Taken together, the results indicate that LPN and LGN axons send differently tuned orientation-selective inputs to V1, even to the overlapping projecting layers.

### Direction Selectivity of the Lateral Posterior Nucleus Axons in V1

**FIGURE 6:**
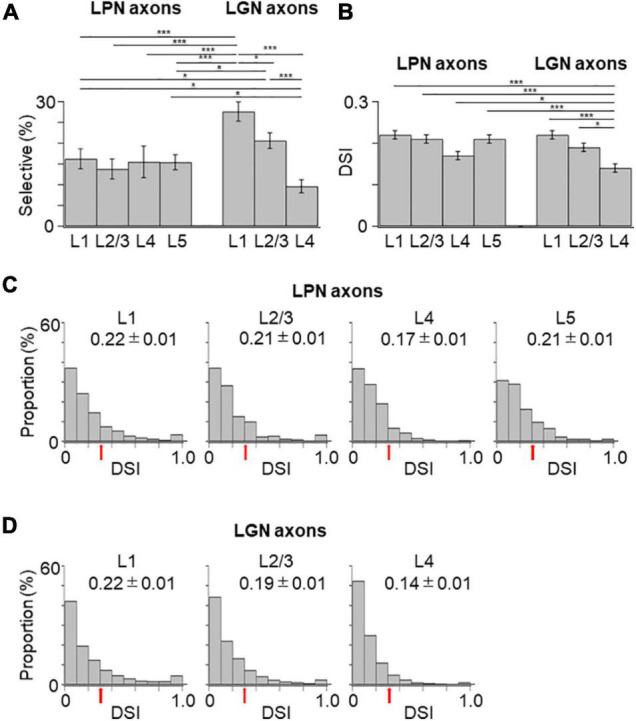
Proportion of direction-selective boutons and distribution of DSI of the LPN and LGN boutons in V1. **(A)** The proportion of direction-selective LPN and LGN boutons. The proportion of the LGN boutons in layer 1 was significantly different from that of the LPN boutons in all the layers (*P* ≤ 0.001, Mann–Whitney *U*-test with Bonferroni correction). The proportion of LGN boutons in layer 2/3 was significantly different from that of LPN boutons in layers 2/3 and 5 (layer 2/3, *p* = 0.032; layer 5, *p* = 0.045, Mann–Whitney *U*-test with Bonferroni correction). The proportion of LGN boutons in layer 4 was significantly different from that of LPN boutons in layers 1 and 5 (layer 1, *p* = 0.023; layer 5, *p* = 0.031, Mann–Whitney *U*-test with Bonferroni correction) and LGN boutons in the remaining layers (*p* ≤ 0.001, Mann–Whitney *U*-test with Bonferroni correction). **(B)** The mean DSI of responsive LPN and LGN boutons. DSI of LGN boutons in the layer 4 was significantly lower than that of LGN boutons in the layer 1, LPN boutons in the layers 1, 2/3, 5 (*P* ≤ 0.001, Mann–Whitney *U*-test with Bonferroni correction) and LGN boutons in the layer 2/3 and LPN boutons in the layer 4 (LGN layer 2/3, *p* = 0.015; LPN layer 4, *p* = 0.041, Mann–Whitney *U*-test with Bonferroni correction). DSI of LGN boutons between the layers 1 and 2/3 (*p* = 1, Mann–Whitney *U*-test with Bonferroni correction) and DSI of LPN boutons among different layers were statistically not different (between layers 1 and 5, *p* = 0.882; other pairs, *p* = 1, Mann–Whitney *U*-test with Bonferroni correction). **(C)** The distribution of DSI of LPN boutons in the layer 1 (left, *n* = 187 boutons from six mice, 11 FOVs), layer 2/3 (second from left, *n* = 93 boutons from six mice, 7 FOVs), layer 4 (second from right, *n* = 81 boutons from five mice, 8 FOVs), and layer 5 (right, *n* = 134 boutons from two mice, 9 FOVs). **(D)** The distribution of DSI of LGN boutons in the layer 1 (left, *n* = 1,954 boutons from 37 mice, 123 FOVs), layer 2/3 (middle, *n* = 1,630 boutons from 36 mice, 97 FOVs), and layer 4 (right, *n* = 931 boutons from 30 mice, 98 FOVs). Arrows show the threshold (DSI = 0.3) for direction-selective boutons. DSI, direction-selective index; LPN, lateral posterior nucleus; LGN, lateral geniculate nucleus; V1, primary visual cortex; FOV, field-of-view. *P ≤ 0.05; ***P ≤ 0.001. The data of LGN axons are re-use from the previous publication (Kondo and Ohki, 2016).

**FIGURE 7:**
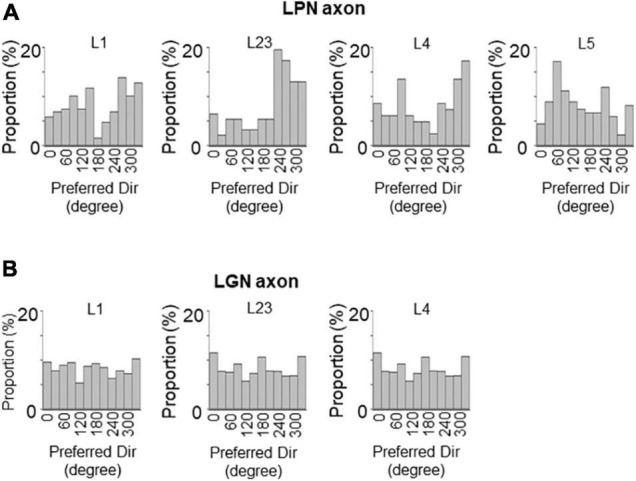
Distribution of preferred directions of LPN and LGN boutons in V1. The distribution of preferred directions of LPN boutons **(A)** and LGN boutons **(B)** in each layer of V1. LPN boutons showed skewed bias from the horizontal to vertical direction, but this bias was less prominent than the orientation bias of LPN boutons. In contrast, LGN boutons showed cardinal bias, which was also less prominent than the orientation bias of LGN boutons. LPN, lateral posterior nucleus; LGN, lateral geniculate nucleus; V1, primary visual cortex. The data of LGN axons are re-use from the previous publication (Kondo and Ohki, 2016).

The proportion of the direction-selective LPN boutons in V1 was similar across the different layers (Figure 6A), while the proportion of the direction-selective LGN boutons projecting to the layer 1 was higher than those projecting to the other layers in V1 (Figure 6A). LGN boutons in the layer 1 showed the highest direction selective proportion, while LGN boutons in the layer 4 had the lowest direction selective proportion among both LGN and LPN boutons (Figure 6A). The DSI of LPN boutons was not significantly different across the different layers (Figures 6B,C). However, DSI of LGN boutons in the layer 1 showed significantly higher selectivity than in the layer 4 (Figures 6B,D). The distribution of the DSI revealed that the boutons in the layer 4 had the lowest mean value, while those in the other layers had similar mean values across both LPN and LGN boutons (Figures 6B,C). The preferred directions of the LPN boutons showed strong bias toward specific directions (Figure 7A). The preferred directions of the LGN boutons showed a weak bias toward the four cardinal axes (Figure 7B). These results suggest that the LPN and LGN axons converging to the same layer carry different direction information.

### High Orientation Selectivity of the Mixed Lateral Geniculate Nucleus and Lateral Posterior Nucleus Axons in V1

**FIGURE 8:**
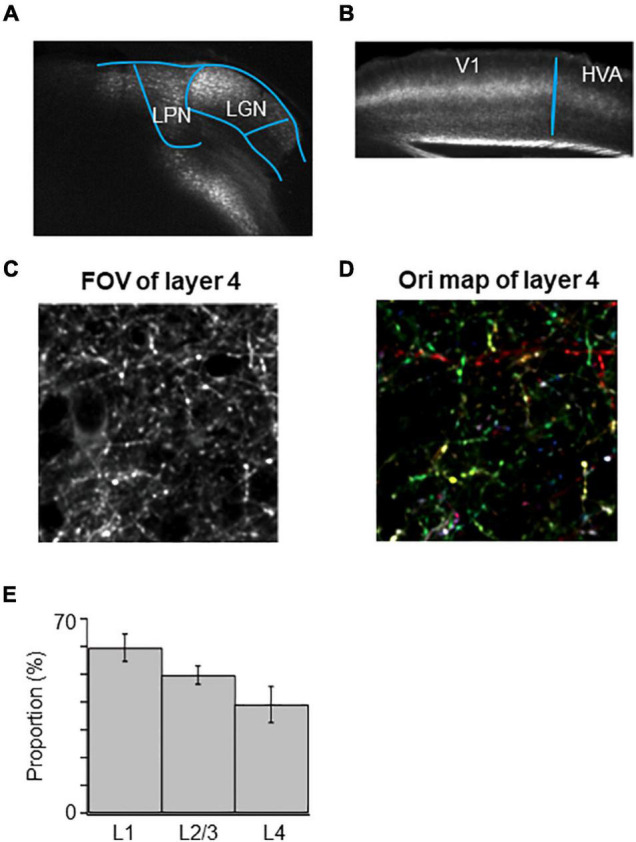
High orientation selectivity of mixed LGN and LPN axons in V1. **(A)** Targeted expression of GCaMP6s to LGN was conducted but expression of GCaMP6s was leaked in LPN. **(B)** Projections of mixed LGN and LPN axons in V1. Projections can be seen not only in the V1 but also in the HVAs. **(C)** A FOV image of the mixed LGN and LPN axons in the layer 4 of V1. **(D)** An orientation color map of the mixed LGN and LPN axons in the layer 4 of V1. Mixed LGN and LPN axons in the layer 4 showed high orientation selectivity. **(E)** The proportion of orientation-selective mixed LGN and LPN boutons in layers 1 (*n* = 941 boutons from 5 mice, 5 FOVs), 2/3 (*n* = 1,106 boutons from 5 mice, 5 FOVs) and 4 (*n* = 1,042 boutons from 5 mice, 5 FOVs). The proportion of orientation-selective mixed LGN and LPN boutons among different layers were statistically not different (between layers 1 and 4, *p* = 0.258; other pairs, *p* = 1, Mann–Whitney *U*-test with Bonferroni correction). LPN, lateral posterior nucleus; LGN, lateral geniculate nucleus; V1, primary visual cortex; FOV, field-of-view.

Because of the difficulty to restrict the expression of GCaMP6s in the small nucleus of LGN, we sometimes encountered the unexpected leakage of GCaMP6s expression in the neighboring LPN (Figure 8A). In these cases, we observed that the mixed LGN and LPN axons project to both V1 and HVAs (Figure 8B). We analyzed the orientation and direction selectivity of mixed LGN and LPN boutons in V1. The orientation color map of layer 4 indicated that the selectivity of the mixed LGN and LPN boutons was higher (Figures 8C,D) than that of LGN boutons in layer 4 (Figure 3B; Kondo and Ohki, 2016). The proportion of orientation-selective boutons were statistically higher in the mixed LGN and LPN boutons (Figure 8E) than in the LGN boutons in all the layers (Figure 4A) (mixed vs. LGN boutons: 58.6 vs. 41.3% in layer 1, *P* = 0.017, unpaired *t*-test; 49.6 vs. 35.6% in layer 2/3, *P* = 0.021, unpaired *t*-test; 38.5 vs. 17.8% in layer 4, *P* ≤ 0.001, unpaired *t*-test). These results suggest that high orientation selectivity can be obtained in the mixed LGN and LPN axons projecting to layer 4 of V1 when the expression of GCaMP6s is targeted to LGN but leaked to LPN.

### Spatial Frequency Selectivity of the Lateral Posterior Nucleus Axons in V1

**FIGURE 9:**
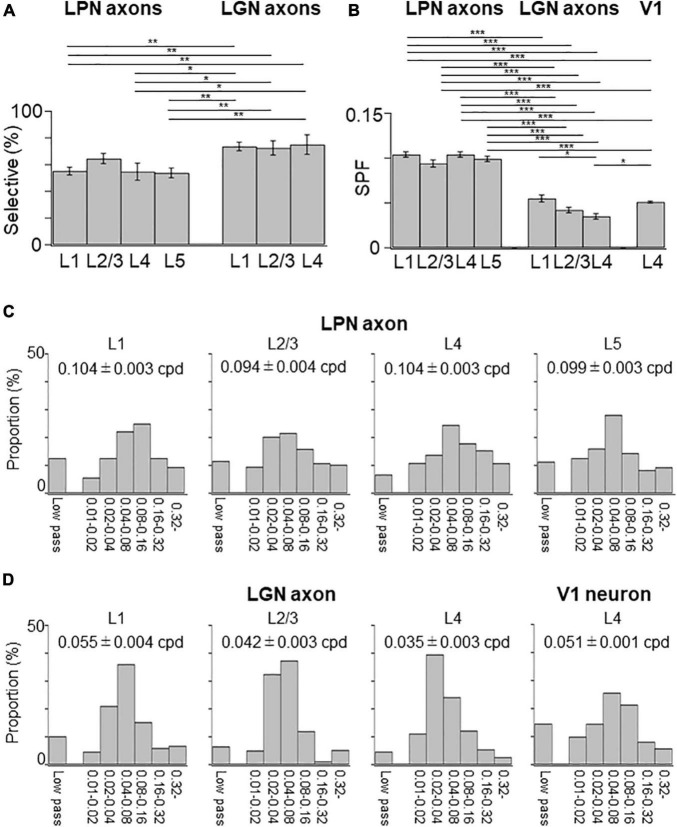
Spatial frequency tuning of LPN and LGN boutons in V1. **(A)** The proportion of SPF-selective LPN and LGN boutons. The proportion of LPN boutons in the layers 1 and 5 was significantly different from that of LGN boutons in all layers (*P* ≤ 0.01, Mann–Whitney *U*-test with Bonferroni correction). The proportion of LPN boutons in layer 4 was significantly different from that of LGN boutons in all layers (layer 1, *p* = 0.024; layer 2/3, *p* = 0.032; layer 5, *p* = 0.047, Mann–Whitney *U*-test with Bonferroni correction). **(B)** The mean preferred SPF of responsive LPN and LGN boutons. Mean preferred frequency of LPN boutons of all the layers had significant difference from LGN boutons of all the layers (*P* ≤ 0.001, Mann–Whitney *U*-test with Bonferroni correction). Mean preferred frequency among LPN boutons had no significant difference (between layers 1 and 2/3, *p* = 0.896; other pairs, *p* = 1, Mann–Whitney *U*-test with Bonferroni correction). Mean preferred frequency of the layer 4 LGN boutons was significantly different from LGN boutons of layer 1 and V1 neurons in layer 4 (LGN boutons in layer 1, *p* = 0.037; V1 neurons in layer 4, *p* = 0.013, Mann–Whitney *U*-test with Bonferroni correction). **(C)** The distribution of the preferred SPFs of LPN boutons in the layer 1 (left, *n* = 893 boutons from six mice, 13 FOVs), layer 2/3 (second from left, *n* = 659 boutons from six mice, 9 FOVs), layer 4 (second from right, *n* = 844 boutons from five mice, 8 FOVs), and layer 5 (right, *n* = 1,000 boutons from three mice, 13 FOVs). **(D)** The distribution of the preferred SPFs of LGN boutons in the layer 1 (left, *n* = 3,817 boutons from 20 mice, 42 FOVs), layer 2/3 (second from left, *n* = 2,669 boutons from 18 mice, 29 FOVs), and layer 4 (second from right, *n* = 5,576 boutons from 20 mice, 55 FOVs), and V1 neurons in the layer 4 (right, *n* = 6,210 neurons from 9 mice, 12 volumes). LPN, lateral posterior nucleus; LGN, lateral geniculate nucleus; V1, primary visual cortex; SPF, spatial frequency; FOV, field-of-view; cpd, cycle per degree. *P ≤ 0.05; **P ≤ 0.01; ***P ≤ 0.001. The data of LGN axons and V1 neurons are re-use from the previous publication (Kondo and Ohki, 2016).

The SPF selectivity of the LPN boutons in V1 was examined and compared with that of the LGN boutons (Figure 9). SPF selectivity of LPN boutons, including those with low-pass tuning (< 0.01 cpd) and high spatial tuning (> 0.32 cpd), was fitted with a difference of Gaussian (DOG) model and examined the range of preferred SPFs. The proportion of the SPF selective LPN boutons was lower than that of the SPF selective LGN boutons, although the difference was not statistically significant (Figure 9A). The mean preferred SPF of the LPN boutons was similar across different layers but significantly higher than that of the LGN boutons (Figures 9B–D). The distribution of the preferred SPFs was broader in the LPN boutons than in LGN boutons in all layers (Figures 9C,D), indicating that the LPN axons had more diverse SPF selectivity than the LGN axons.

## Discussion

In this study, *in vivo* two-photon calcium imaging of LPN axons projecting to V1 was performed to examine their orientation/direction and SPF selectivity, and the findings were compared with the results from our previous study of LGN axons (Kondo and Ohki, 2016). Collectively, the data show that LPN axons project mainly to the layers 1 and 5 and, to a lesser extent, layers 2/3 and 4, while LGN axons project mainly to the layer 4 and, to a lesser extent, layers 1 and 2/3. LPN axons send orientation- and direction-selective inputs to all the layers, while LGN axons send strong orientation- and direction-selective inputs to the layer 1 and, to a lesser degree, layer 4. Both LPN and LGN axons send SPF-selective inputs to all the layers of V1. However, the SPF selectivity of LPN axons is more diverse than that of LGN axons.

The visual information carried by the LPN neurons has multiple origins. The data from our retrograde labeling study, along with those of other previous study (Bennett et al., 2019), have shown that the LPN receives feedforward inputs from the SC. Furthermore, a transsynaptic retrograde labeling study has shown that the LPN neurons projecting to V1 receive inputs from the SC neurons (Fang et al., 2020). Thus, the LPN activity projecting to V1 could be partly derived from the SC. Many neurons in the superficial layers of the SC (sSC) in mice are tuned for orientation and motion directions (Wang et al., 2010; Gale and Murphy, 2014; Ahmadlou and Heimel, 2015; Feinberg and Meister, 2015; De Franceschi and Solomon, 2018). Therefore, the orientation and direction selectivity of the LPN axons that we observed in this study is potentially derived from the response selectivity of the sSC neurons. Orientation selectivity is heterogeneously distributed in the mouse sSC and is dependent on the retinotopic position (Ahmadlou and Heimel, 2015; Feinberg and Meister, 2015; Kasai and Isa, 2021). Although we did not measure the retinotopic position during the recording, we usually recorded activity of LPN axons at the similar stereotaxic position in V1. Therefore, biased orientation selectivity observed in this study may reflect the orientation bias of sSC neurons. Given that the sSC receives inputs from the retinal ganglion cells, the characteristic direction selectivity of the LPN neurons can be further traced back to the retinal direction-selective ganglion cells (Shi et al., 2017; Fang et al., 2020).

Another potential origin of the visual information carried by the LPN axons could be V1 or HVA. The LPN has been shown to be reciprocally connected to V1 and HVAs (Bennett et al., 2019; Blot et al., 2021). Thus, LPN activity may also be driven by these feedback visual inputs from the lower and higher visual cortices. Previous studies have shown that lesions or inhibition of the V1 neurons alters neuronal activity in the anterior subregion of the LPN (Bennett et al., 2019), but that it does not largely alter the selectivity of the orientation and direction responses in the sSC neurons (Wang et al., 2010; Ahmadlou and Heimel, 2015). The V1 is connected to the SC unidirectionally and the SC is connected to the LPN unidirectionally as well. Thus, V1-SC-LPN pathway can also be a potential origin of the visual information carried by the LPN axons, but the above results suggest that this pathway may be not so important as reciprocal pathways of the LPN and V1 or LPN and HVAs.

Taken together, the orientation- and direction-selective responses of the LPN axons may originate from either feedforward sSC inputs to the LPN derived from the retinal direction-selective ganglion cells (Shi et al., 2017; Fang et al., 2020) or feedback visual inputs from V1 or the HVAs to the LPN (Zhou et al., 2017; Bennett et al., 2019; Juavinett et al., 2020; Blot et al., 2021). Future studies examining the activity of the LPN axons in V1 under the suppression of the visual cortices or SC will clarify the origin of the visual information carried by the LPN axons in V1.

Our previous study has shown the low orientation selectivity in the LGN axons projecting to the layer 4 of V1 (Kondo and Ohki, 2016), which is different from the results of other studies (Sun et al., 2016; Zhuang et al., 2021). The reason for the discrepancy between these studies could be the differences in experimental conditions, particularly, the type of promoter used to express the genetically encoded calcium indicator protein, GCaMP6s. The CAG promoter that we used in our studies (Kondo and Ohki, 2016) is one of ubiquitous promoters and labels a large population of neurons (Jackson et al., 2016). Therefore, our samples may represent a larger population of LGN neurons than other two groups.

Another difference between our study and the two studies is the optics of two-photon microscope. Sun et al. (2016) and Zhuang et al. (2021) used adaptive optics (AO) to improve the distortion of the fluorescence signal due to optical aberrations in the recordings from the deep cortical layers. A previous study showed that the sample-induced aberration during the *in vivo* brain imaging is mostly a spherical aberration (Ji et al., 2012). The spherical aberration can be corrected effectively by adjusting the axial position of the objective lens (Kondo and Ohki, 2016). Since our recordings were done by this method, the effect of sample-aberration was minimized without AO. Indeed, Zhuang et al. (2021) tested this possibility and found that AO improves the signal intensity of visual responses, but does not affect the calculation of the orientation selectivity of axonal boutons in layer 4. In the present study, we found that the LPN axons projecting to the layer 4 of V1 have high orientation selectivity, suggesting that our correction method is enough to see the orientation selectivity, and the low orientation selectivity of the LGN axons projecting to V1 (Kondo and Ohki, 2016) was not due to the sample aberration.

Finally, we observed that LPN axons projecting to the layer 4 of V1 had high orientation selectivity, while LGN axons projecting to the layer 4 of V1 had low orientation selectivity. Because of the difficulty to restrict the expression of GCaMP6s in the small nucleus of LGN, unexpected leakage of GCaMP6s expression in the neighboring LPN could happen. If it is the case, recordings of the mixed LGN and LPN axons may result in the higher proportion of orientation-selectivity in the layer 4 (Figure 8) than the LGN axons.

The orientation-selectivity of LPN axons observed here (mean gOSI = 0.27 ∼ 0.38, gOSI = 0.27 in layer 1) were higher than those of LPN neurons (Durand et al., 2016: mean OSI = 0.22, Fang et al., 2020: mean gOSI = 0.17) but comparable with that of LPN axons in V1 (Roth et al., 2016: mean OSI = 0.38 in layer 1) reported previously (gOSI becomes usually lower than OSI). On the other hand, the DSI was similar between our study and these studies (Our study: mean DSI = 0.17 ∼ 0.22, Durand et al., 2016: mean DSI = 0.34, Roth et al., 2016: mean DSI = 0.27 in layer 1). The location of LPN neurons targeting specific brain regions are localized within the LPN (Bennett et al., 2019, e.g., neurons targeted to V1 and ventral HVA locate mainly in the anterior part of LPN, while neurons targeted to dorsal HVA locate mostly in posterior part of LPN). The stereotaxic coordinates of the recording sites of LPN neurons (Durand et al., 2016, 2.3 mm posterior from Bregma; Fang et al., 2020, 2.2 ∼ 2.3 mm posterior from Bregma) were more posterior than our AAV injected site (our study, 1.8 mm posterior from Bregma). Therefore, the difference in the orientation selectivity between this study and the previous studies may arise from the difference in the population of recorded neurons, where neurons targeting dorsal HVA may be less orientation selective than those targeting V1.

Our recordings of the axonal activities of the LPN and LGN neurons were carried out under the light anesthesia (0.2% isoflurane sedated by chlorprothixene). Since LPN receives inputs from various brain regions such as superior colliculus (SC), higher visual areas, amygdala, and other sensory areas (Zhou et al., 2017; Bennett et al., 2019), these brain regions may affect the response properties of LPN neurons depending on the brain states (Durand et al., 2016, e.g., anesthesia or wakefulness). It is recently reported that the functional organization of orientation-selective SC neurons (Ahmadlou and Heimel, 2015; Feinberg and Meister, 2015; Chen et al., 2021) were dynamically modulated by the brain states (Kasai and Isa, 2021). This state-dependent difference in the orientation-selectivity of SC neurons (Kasai and Isa, 2021) and in neuronal activities of other brain regions (Poulet and Crochet, 2019; Hua et al., 2020) may accordingly affect the orientation-selectivity of LPN neurons.

It is reported that the LPN is reciprocally connected with many cortical areas, such as visual, auditory, and somatosensory cortices (Zhou et al., 2017; Bennett et al., 2019) and possibly integrates multisensory information within the LPN circuit. It has been also suggested that the LPN may function as a hub providing indirect route to transfer sensory information from one cortex to other (Sherman, 2016). Thus, the unique anatomical position of the LPN may have important functional roles for the integration and modulation among the multisensory brain circuits (Chou et al., 2020; Fang et al., 2020). Modulatory function of other higher order thalamus has been also reported in the primary somatosensory cortex, showing that the posterior medial (POm) nucleus of the thalamus enhances the amplitude of whisker deflection (Gharaei et al., 2020).

The orientation- and direction-selective inputs from LPN to different layers in V1 may have different roles in each layer. In layer 4, neurons show sharp orientation selectivity (Niell and Stryker, 2008; Kondo et al., 2016). The orientation selectivity of V1 neurons in layer 4 may be derived from the orientation-non-selective LGN inputs (Hubel and Wiesel, 1962; Lien and Scanziani, 2013) and the orientation-selective LPN inputs may further contribute to the orientation selectivity of V1 neurons in layer 4. The LPN axons send more diverse SPF information than LGN axons to V1. Since the V1 neurons show more diverse SPF selectivity (Niell and Stryker, 2008; Kondo et al., 2016) than LGN axons, diverse SPF selectivity of LPN inputs may contribute to the diverse selectivity of V1 neurons.

## Data Availability Statement

The data that support the findings of this study and all custom code used in this study are available from the corresponding author upon reasonable request.

## Ethics Statement

The animal study was reviewed and approved by the University of Tokyo Animal Care and Use Committee.

## Author Contributions

SK and KO designed the research and wrote the manuscript. SK and YK performed the experiments and analyzed the data. All authors contributed to the article and approved the submitted version.

## Conflict of Interest

The authors declare that the research was conducted in the absence of any commercial or financial relationships that could be construed as a potential conflict of interest.

## Publisher’s Note

All claims expressed in this article are solely those of the authors and do not necessarily represent those of their affiliated organizations, or those of the publisher, the editors and the reviewers. Any product that may be evaluated in this article, or claim that may be made by its manufacturer, is not guaranteed or endorsed by the publisher.
