# Supplementary material for: Response Selectivity of the Lateral Posterior Nucleus Axons Projecting to the Mouse Primary Visual Cortex
Source: Front Neural Circuits. 2022 Feb 28;16:825735. doi: 10.3389/fncir.2022.825735 (PMC8918919; doi:10.3389/fncir.2022.825735)
Supplement: Supplementary file 1 [file Data_Sheet_1.pdf]

Supplementary Information

Response selectivity of the lateral posterior nucleus axons projecting to the mouse primary visual cortex

Kondo S, Kiyohara Y and Ohki K

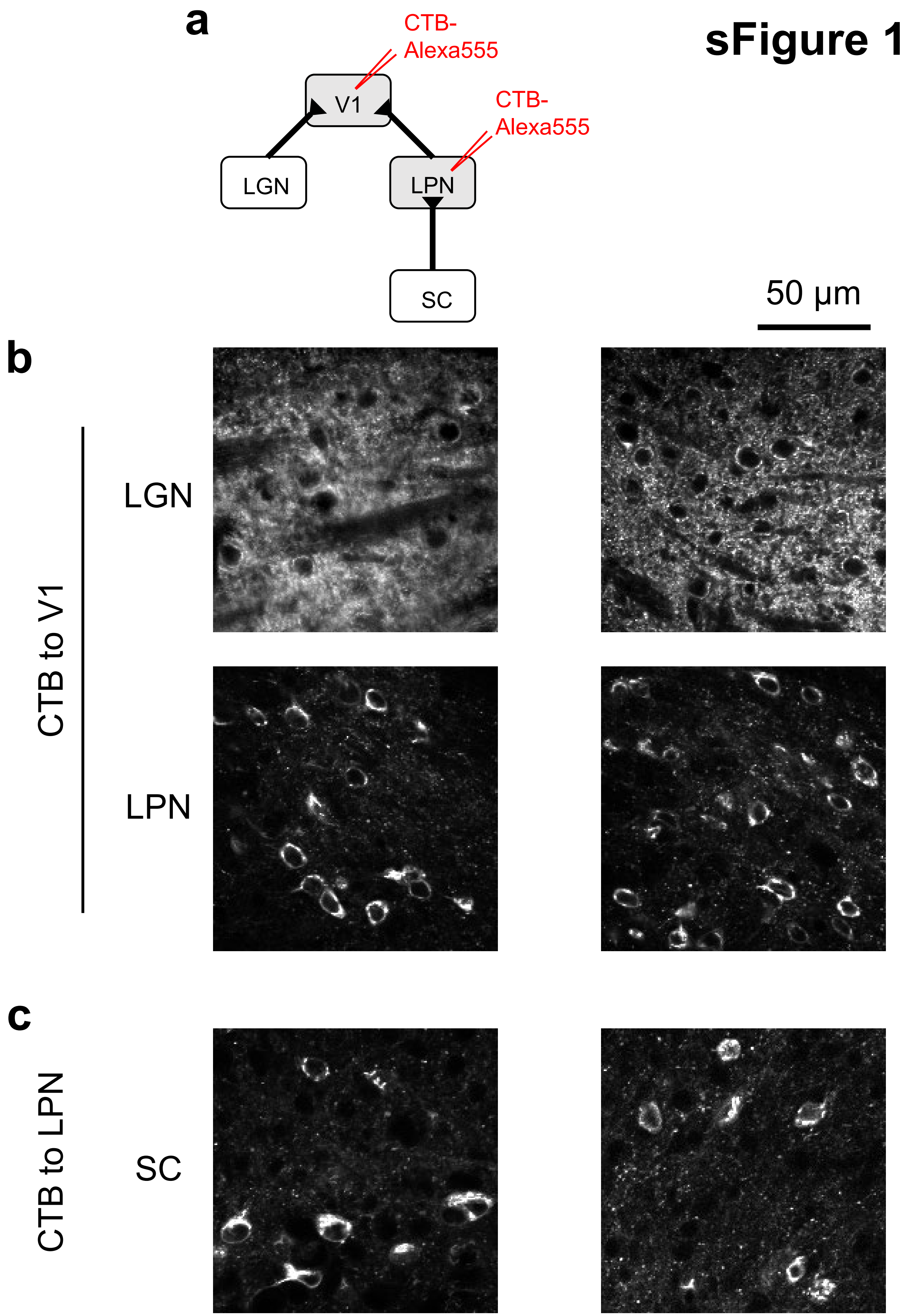
sFigure 1
a
CTB-
Alexa555
V1
CTB-
Alexa555
LGN
LPN
SC
50 µm
b
CTB to V1
LGN
LPN
c
CTB to LPN
SC

Supplementary Figure 1. High magnified photographs showing retrogradely labeled neurons.

(a) A retrograde labeling by CTB injection into either V1 or LPN as shown in Figure 1. (**b**) High magnified photographs from the retrogradely labeled area of LGN (Figure 1b lower panel) and LPN (Figure 1c lower panel) were taken using confocal microscope. The morphology of retrogradely labeled LGN and LPN neurons can be seen. (**c**) High magnified photographs from the retrogradely labeled area of sSC (Figure 1c lower panel) were taken using confocal microscope. The morphology of retrogradely labeled sSC neurons can be seen. LGN, lateral geniculate nucleus; LPN, lateral posterior nucleus; CTB, cholera toxin subunit B; sSC, superficial layers of the superior colliculus
